# Supplementary material for: Binary-level data dependence analysis of hot execution regions using abstract interpretation at runtime
Source: PLoS One. 2020 Apr 9;15(4):e0230904. doi: 10.1371/journal.pone.0230904 (PMC7144976; doi:10.1371/journal.pone.0230904)
Supplement: Attachment — Submitted filename: Response to Reviewers.pdf [file pone.0230904.s001.pdf]

Reply letter to the reviewer comments on the paper titled

“Binary-level data dependence analysis of hot execution regions using abstract interpretation at runtime”

First of all, we would like to express our gratitude to the associate editor and anonymous reviewers for their careful reviews and valuable suggestions. The comments are well taken and, accordingly, the manuscript has been revised carefully. Reviewers’ comments are represented using **Bold Red Font.** All revisions in the manuscript have been marked with **Bold Blue Font** color. Our point-to-point responses to the comments are listed as follows.

Responses to Reviewer

Reviewer #1:

1- **Why is the evaluation limited to Polybench? What is the rationale for evaluating the proposed work only on linear codes?Why were some of the benchmarks in Polybench exclude?**

a. **Why is the evaluation limited to Polybench?**

- **Response:** We would like to thank the anonymous reviewer for careful review of our work. Please note that, Polybench is Polyhedral Benchmark suite. Each Polybench kernel could be used as a single file for instrumentation. Moreover, the execution of the kernel would be initialized with non-null data and live-out data dump. Also, the kernels contain parametric loop bounds to be utilized for general-purposed implementation. We have used Polybench kernels because they contain a bunch of instructions which could be handled by our system during runtime. Therefore, the experiments would be applied on other benchmarks, but we will need to study their kinds of instructions and how they would be handled using our technique. However, the applied instructions’ results show that our technique have no problem to be applied and implemented in other more complex kernels. We have addressed this comment in the revised manuscript by adding few sentences in (page 15, Section 5, lines 635-644) and for easy of your reference, we write it down here:

“**Furthermore, we study the main results produced from the proposed method on Polybench [35]. Polybench is called from the Polyhedral Benchmark suite. It is used to uniformize the kernels execution and monitoring. Moreover, the kernels of Polybench are used as a single file to perform the kernel instrumentation. Also, the kernels would run with non-null data initialization and live-out data dump. The loop bounds in each kernel are parametric to be utilized for general-purpose implementation. Moreover, the method impact is studied in two selected programs from SPEC CPU 2006 in terms of the overhead.**”

b. **What is the rationale for evaluating the proposed work only on linear codes?**

- **Response:** Please note that our work monitors the behavior of the addresses not data. Therefore, the system would target the linear and non-linear codes. In this paper, our

system is applied on linear codes to preserve the primary results because of instruction operations' types. Moreover, we have added the overhead of applying our system to two kernels from SPEC, 401.gcc and 403.bzip2. This response is reflected in the revised manuscript (page 4, section 1, lines 126-136). For easy of your reference, the modified paragraphs are written as:

"Furthermore, our technique detects dependence correctly in the case of pointer aliasing which refers to existing of two pointers with the same value. If a pointer aliasing occurs, this may cause a dependence during the parallelized execution. Also, our system has no information about the fact that loop bounds/array indices are linear or not. This fact could be leveraged by any optimization that needs this information. Therefore, our approach does not need to build a polyhedral model. The main focus is on ranges which are determined during runtime by observing the addresses included in the hot trace. The method is able to handle the affine and semi-affine codes because of the dynamic monitoring of the addresses of the program points during runtime. Thus, our proposed technique still proves that a loop is free of dependence."

- Moreover, we explain this issue at (page 5, section 2.1, line 184-188):

"Our approach could monitor the main behavior of the addresses. This monitoring would be utilized to convert the used addresses concrete domain into abstract domain. This abstract domain would be applied to extract any dependence in all types of accesses, direct or indirect. Also, the system would be applied for linear and non-linear systems."

c. **Why were some of the benchmarks in Polybench excluded?**

- **Response:** We have added the excluded kernels as in the table true/false positives and the overheads are computed.
- This concept is clearly explained in the paper at (page 15-16, Section 5, lines 640-655) and for easy of your reference, here we mention regarding this comment as follows:

"Our approach is examined by linear algebra kernels for this initial study. All used kernels include dependence in the hottest extracted function in the code by Padrone lightweight profiler. The initial study results are demonstrated in Table 2 and Fig. 2.

Fig. 2 illustrates the overhead as a main metric for dependence detection techniques. The overhead is computed as the difference between the execution time of the kernels executed through our proposed approach and the execution time of the original running on basic kernels. The overhead is partitioned as Padrone overhead and our proposed method overhead. The Padrone overhead is ranged from about 1% to 14.3%. This overhead includes the Padrone profiling and extracting the hot function during runtime. Also, the overall overhead of *401.gcc* and *403.bzip2* kernels are about *18%*. On the other

hand, our proposed analysis technique overhead is between 0.2% to 4.88%. Therefore, the overall overhead is mostly less than *10%* which could be considered comparatively very low percentage. However, we could notice that the overhead increases on the other kernels. This increase is a result of the larger number of program instructions which are checked during runtime. This overhead will be diminished by applying the speculative parallelization in the full speculative system."

- Figure 2 is modified as shown below:

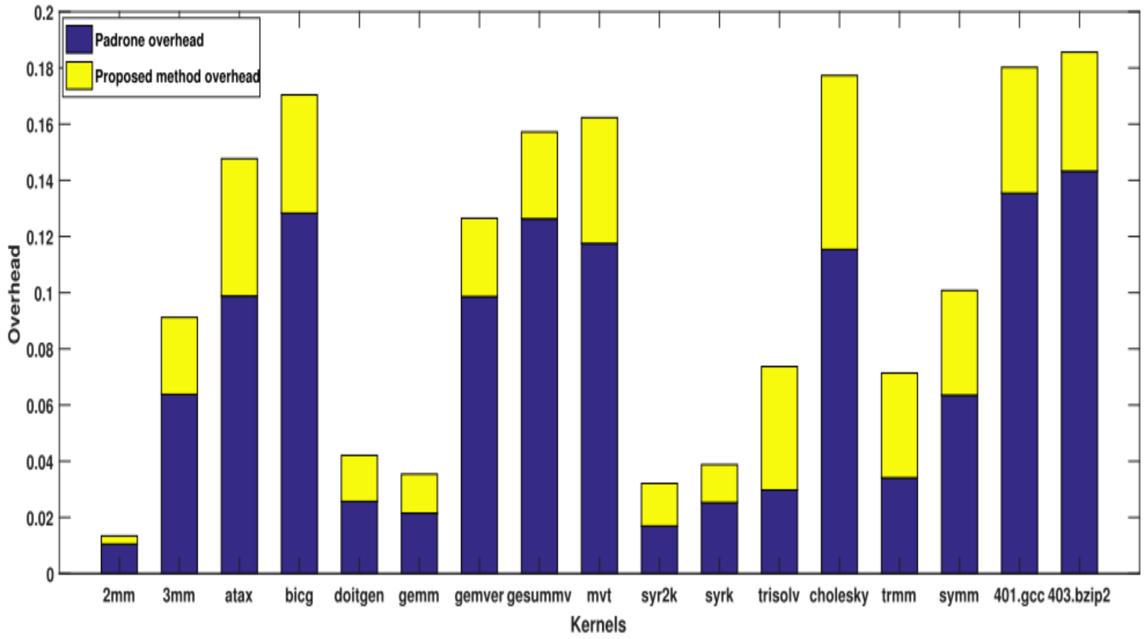

- Also, Table 2 is provided after modification in <u>(page 16)</u> and it is included below for easy of your reference.

Table 2. Dependence Detected in Kernels of Polybench by Our Method and Static AI (True and False Positives)

Kernel	True Positives (Our Approach)	False Positives (Our Approach)	True Positives (Static AI)	False Positives (Static AI)
2mm	1	0	1	2
3mm	1	0	1	2
bicg	2	0	2	2
gesummv	1	0	1	1
gemm	1	0	1	2
mvt	1	0	1	2
syrk	1	0	1	1
syr2k	2	0	2	1
atax	1	2	1	2
gemver	1	0	1	1
doitgen	1	2	1	2
trisolv	2	1	2	2
cholesky	3	1	3	2
trmm	1	1	1	2
symm	3	1	3	2

2- **The authors should "clearly" specify the kind of code this framework can handle. Is this work compatible with non-linear codes? If yes, evaluate the proposed approach on SPEC benchmarks; If not why? The restriction of the approach and the implementation should be separately discussed.**

a. **The authors should "clearly" specify the kind of code this framework can handle.**

- **Response:** Thank you for your question. Our method could handle all types of code because the system would monitor all program points existing in the hot trace of the hot function. We have applied the method mainly in the instructions been used in Polybench kernels which is a polyhedral benchmark to get results of the correctness and overhead produced. We have mentioned this clearly in our manuscript at (page 5, Section 2.1, line 184-188). We put this paragraph below for easy of your reference:

"Our approach could monitor the main behavior of the addresses. This monitoring would be utilized to convert the used addresses concrete domain into abstract domain. This abstract domain would be applied to extract any dependence in all types of accesses, direct or indirect. Also, the system would be applied for linear and non-linear systems."

- And to clearly illustrate the difference between our proposed approach and other related approaches, we added the following paragraph at (page 4, section 1, line 123-136):

"Our work differs mainly from the related work in that we conduct analysis at runtime. This provides much accurate dependence detection. Moreover, the method is automatic and dynamic which does not need any additional directives which restricts the speculative parallelization to re-execute the code. Furthermore, our technique detects dependence correctly in the case of pointer aliasing which refers to existing of two pointers with the

same value. If a pointer aliasing occurs, this may cause a dependence during the parallelized execution. Also, our system has no information about the fact that loop bounds/array indices are linear or not. This fact could be leveraged by any optimization that needs this information. Therefore, our approach does not need to build a polyhedral model. The main focus is on ranges which are determined during runtime by observing the addresses included in the hot trace. The method is able to handle the affine and semi-affine codes because of the dynamic monitoring of the addresses of the program points during runtime. Thus, our proposed technique still proves that a loop is free of dependence."

- Also, the response to such comment is clearly addressed by adding the following sentences in (page 10 , section 4, line 407-412) :

"Our system studies the behavior of each address included in the hot trace. This hot trace consists of the hot consecutive program points that are usually executed at runtime. Also, the preserved hot trace would let the analysis compute the loop abstract intervals for bounds. The program points, which are not involved in the current hot trace, will not be analyzed. Therefore, our approach could elicit the dependent instruction pairs which are subsequent of the analyzed function hot trace."

b. **Is this work compatible with non-linear codes? If yes, evaluate the proposed approach on SPEC benchmarks; If not why? The restriction of the approach and the implementation should be separately discussed.**

- **Response:** We would like to thank you for this note. As mentioned before, our system could be compatible with linear and non-linear codes. We mainly implemented the instructions used by Polybench, however we have handled two programs in SPEC Benchmarks (401.gcc and 403.bzip2) in order to prove our system ability to be applied with non-linear codes. We studied the SPEC two programs in terms of overhead. The binary code of the two kernels includes huge number of instructions. Therefore, the accuracy would be studied with the speculative full system. Thus, we have added the correctness and the more complicated kernels as future work.
- This is mentioned clearly in the paper at (page 4, section 1, line 123-136), and for easy of your reference we write it down here as:

"Our work differs mainly from the related work in that we conduct analysis at runtime. This provides much accurate dependence detection. Moreover, the method is automatic and dynamic which does not need any additional directives which restricts the speculative parallelization to re-execute the code. Furthermore, our technique detects dependence correctly in the case of pointer aliasing which refers to existing of two pointers with the same value. If a pointer aliasing occurs, this may cause a dependence during the parallelized execution. Also, our system has no information about the fact that loop

bounds/array indices are linear or not. This fact could be leveraged by any optimization that needs this information. Therefore, our approach does not need to build a polyhedral model. The main focus is on ranges which are determined during runtime by observing the addresses included in the hot trace. The method is able to handle the affine and semi-affine codes because of the dynamic monitoring of the addresses of the program points during runtime. Thus, our proposed technique still proves that a loop is free of dependence."

- Also, this is explained in the paper at (page 5, section 2.1, line 184-188), and for easy of your reference we write it down here as:

"Our approach could monitor the main behavior of the addresses. This monitoring would be utilized to convert the used addresses concrete domain into abstract domain. This abstract domain would be applied to extract any dependence in all types of accesses, direct or indirect. Also, the system would be applied for linear and non-linear systems."

- Moreover, this issue is discussed in the paper at (page 15-16, section 5, line 640-655), and for easy of your reference we write it down here as:

"Our approach is examined by linear algebra kernels for this initial study. All used kernels include dependence in the hottest extracted function in the code by Padrone lightweight profiler. The initial study results are demonstrated in Table 2 and Fig. 2.

Fig. 2 illustrates the overhead as a main metric for dependence detection techniques. The overhead is computed as the difference between the execution time of the kernels executed through our proposed approach and the execution time of the original running on basic kernels. The overhead is partitioned as Padrone overhead and our proposed method overhead. The Padrone overhead is ranged from about 1% to 14.3%. This overhead includes the Padrone profiling and extracting the hot function during runtime. Also, the overall overhead of 401.gcc and 403.bzip2 kernels are about 18%. On the other hand, our proposed analysis technique overhead is between 0.2% to 4.88%. Therefore, the overall overhead is mostly less than 10% which could be considered comparatively very low percentage. However, we could notice that the overhead increases on the other kernels. This increase is a result of the larger number of program instructions which are checked during runtime. This overhead will be diminished by applying the speculative parallelization in the full speculative system."

- Also, Figure 2 is provided in the paper as below:

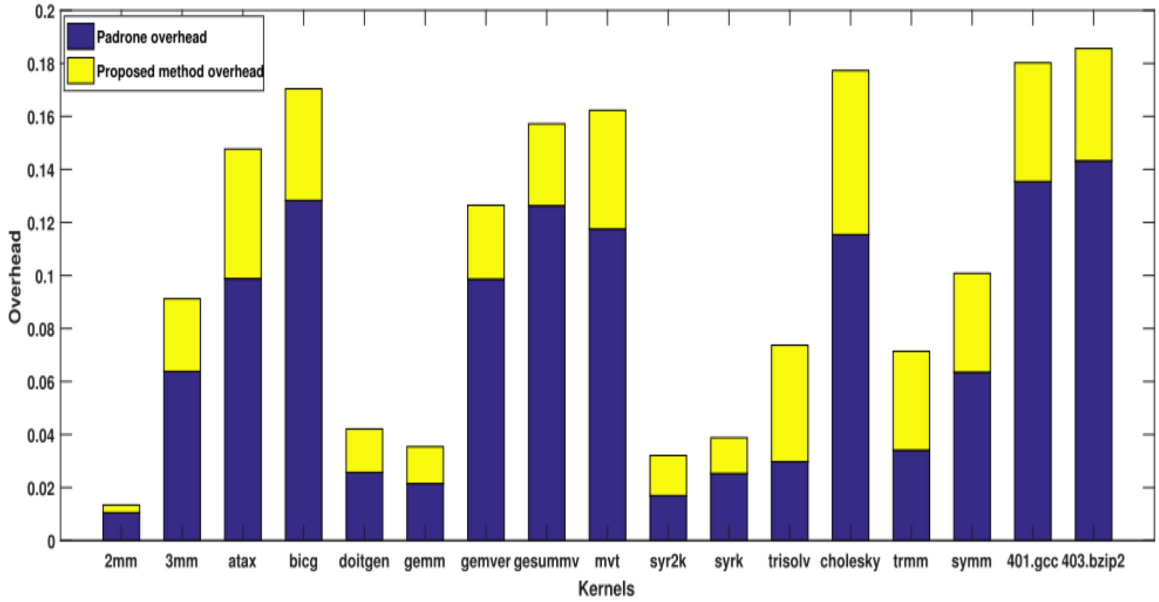

3- **How important is the notion of "loop iterators" and "loop bounds" for this paper? Is this work compatible with codes with indirect accesses?**

a. **How important is the notion of "loop iterators" and "loop bounds" for this paper?**

- **<u>Response:</u>** Thank you for giving us a chance to clarify this issue. Our approach monitors the actual values written to or read from the loop iterator during runtime. According to the monotonicity of these actual values, the loop bounds are computed using widening. In our paper, our approach watches the behavior of the addresses and the monotonicity of the loop iterators. Therefore, our system could compute the range of addresses being used by each program point by widening the loop iterator. The computed range would be the loop bound regardless the loop is linear or non-linear.
- Therefore, the manuscript is updated accordingly in <u>(page 10 , section 4, line 407-412)</u> :

"Our system studies the behavior of each address included in the hot trace. This hot trace consists of the hot consecutive program points that are usually executed at runtime. Also, the preserved hot trace would let the analysis compute the loop abstract intervals for bounds. The program points, which are not involved in the current hot trace, will not be analyzed. Therefore, our approach could elicit the dependent instruction pairs which are subsequent of the analyzed function hot trace."

b. **Is this work compatible with codes with indirect accesses?**

- **<u>Response:</u>** Thanks for this notice. Our technique works on the used addresses during runtime. The addresses have been converted from concrete domain to abstract domain. The abstract domain could be utilized to detect dependence by intersecting between each pair of program points 'abstract intervals, regardless the type of accesses. Therefore, our work would be compatible with the programs with indirect accesses. The main limitation for

indirect access is where the computed abstract interval of addresses is [-inf, inf], therefore not all reads and write will be captured.

- This is clearly explained in the paper at (page 5, section 2.1, line 184-188) as follows:

"Our approach could monitor the main behavior of the addresses. This monitoring would be utilized to convert the used addresses concrete domain into abstract domain. This abstract domain would be applied to extract any dependence in all types of accesses, direct or indirect. Also, the system would be applied for linear and non-linear systems."

- Also, this response is discussed in the paper at (page 4, Section 1, lines 126-136) as follows:

"Furthermore, our technique detects dependence correctly in the case of pointer aliasing which refers to existing of two pointers with the same value. If a pointer aliasing occurs, this may cause a dependence during the parallelized execution. Also, our system has no information about the fact that loop bounds/array indices are linear or not. This fact could be leveraged by any optimization that needs this information. Therefore, our approach does not need to build a polyhedral model. The main focus is on ranges which are determined during runtime by observing the addresses included in the hot trace. The method is able to handle the affine and semi-affine codes because of the dynamic monitoring of the addresses of the program points during runtime. Thus, our proposed technique still proves that a loop is free of dependence."

- The limitation for indirect access is explained in the paper at (page 13, Section 4.3, lines 531-534) as follows:

"For memory indirect accesses, if the current executing program point generates [-inf, inf] results for the needed address for read or write, our system does not model memory locations; the whole memory is abstracted as one interval. Therefore, for this case, most reads and writes related to this instruction may not be captured."

4.1- Compare the scope of this work w.r.t Apollo. Apollo can support affine and semi-affine codes. Can this framework handle the same?

a. Compare the scope of this work w.r.t Apollo.

- **Response:** Thank you for your comment. Apollo extracts the affine expressions of the memory accesses for nested loops regardless the type of memory access. The technique builds a polyhedral model to be used by the speculative parallelizer. Apollo needs preparations to begin analysis. Moreover, it requires re-compilation. Moreover, the system is limited to Polyhedral model.

b. Apollo can support affine and semi-affine codes. Can this framework handle the same?

- **Response:** Apollo could detect the affine memory accesses in nested loops. Our method would detect the memory accesses for each program point. Therefore, it would be able to detect the dependence in nested loops for affine memory accesses. However, our method does not need re-compilation or re-execution.

- This part is highlighted in the modified manuscript as shown at (page 3, section 1, lines 101-111), and for easy of your reference, we write it down here:

"Caamano et al. [14] proposed an automatic speculative polyhedral loop optimizer. On-the-fly polyhedral optimizations are applied on nested loops. These loops include memory accesses patterns and its control flow which may not computed at runtime. Apollo detects the affine expressions of the memory accesses for nested loops to be applied to speculative transformation [15]. Thus, the technique could be applied on any kind of nests loops which may contain memory accesses through pointers or indirect accesses as well as the unpredictable control. The method constructs a predictive polyhedral model to be utilized by speculative optimization. The system requires preparations to get the computed perfect version. These preparation should lead the system to re-compile. Moreover, Apollo technique is limited to the Polyhedral model."

- Also, the main difference is explained in (page 4, Section 1, 123-136):

"Our work differs mainly from the related work in that we conduct analysis at runtime. This provides much accurate dependence detection. Moreover, the method is automatic and dynamic which does not need any additional directives which restricts the speculative parallelization to re-execute the code. Furthermore, our technique detects dependence correctly in the case of pointer aliasing which refers to existing of two pointers with the same value. If a pointer aliasing occurs, this may cause a dependence during the parallelized execution. Also, our system has no information about the fact that loop bounds/array indices are linear or not. This fact could be leveraged by any optimization that needs this information. Therefore, our approach does not need to build a polyhedral model. The main focus is on ranges which are determined during runtime by observing the addresses included in the hot trace. The method is able to handle the affine and semi-affine codes because of the dynamic monitoring of the addresses of the program points during runtime. Thus, our proposed technique still proves that a loop is free of dependence."

4.2- Can this work be used to build the Polyhedral model so that more advanced loop transformations can be applied (Apollo does something like this)? Or is the work just directed towards speculative parallelization /vectorization

- **Response:** We would like to thank you for this note. Our proposed technique detects the existing dependence. This would be done by observing the addresses used by each instruction at runtime. Dependence detection is applied regardless the type of memory accesses or loop type. Therefore, we would not need to build a Polyhedral model. Moreover, the information of loop type is not required for our approach. To alleviate any further confusion the manuscript was updated in (page 4, Section 1, 123-136) as follows:

"Our work differs mainly from the related work in that we conduct analysis at runtime. This provides much accurate dependence detection. Moreover, the method

is automatic and dynamic which does not need any additional directives which restricts the speculative parallelization to re-execute the code. Furthermore, our technique detects dependence correctly in the case of pointer aliasing which refers to existing of two pointers with the same value. If a pointer aliasing occurs, this may cause a dependence during the parallelized execution. Also, our system has no information about the fact that loop bounds/array indices are linear or not. This fact could be leveraged by any optimization that needs this information. Therefore, our approach does not need to build a polyhedral model. The main focus is on ranges which are determined during runtime by observing the addresses included in the hot trace. The method is able to handle the affine and semi-affine codes because of the dynamic monitoring of the addresses of the program points during runtime. Thus, our proposed technique still proves that a loop is free of dependence."

5- Does the overhead reported in Figure 2 include profiling and function extraction overheads? It is better to split the overhead of each component and redraw Figure 2 as a stacked bar chart.

- **Response:** Thank you for your comment. The figure is updated with the overhead caused by Padrone profiling and hot function extraction overheads. Moreover, the overhead from the dynamic data dependence is presented. Therefore, the overall overhead is shown as the total of the above-mentioned overheads. The manuscript is updated in (page 16, Section 5, 643-647) as follows:

"Fig. 2 illustrates the overhead as a main metric for dependence detection techniques. The overhead is computed as the difference between the execution time of the kernels executed through our proposed approach and the execution time of the original running on basic kernels. The overhead is partitioned as Padrone overhead and our proposed method overhead."

- Figure 2 is updated with stack bar chart as follows:

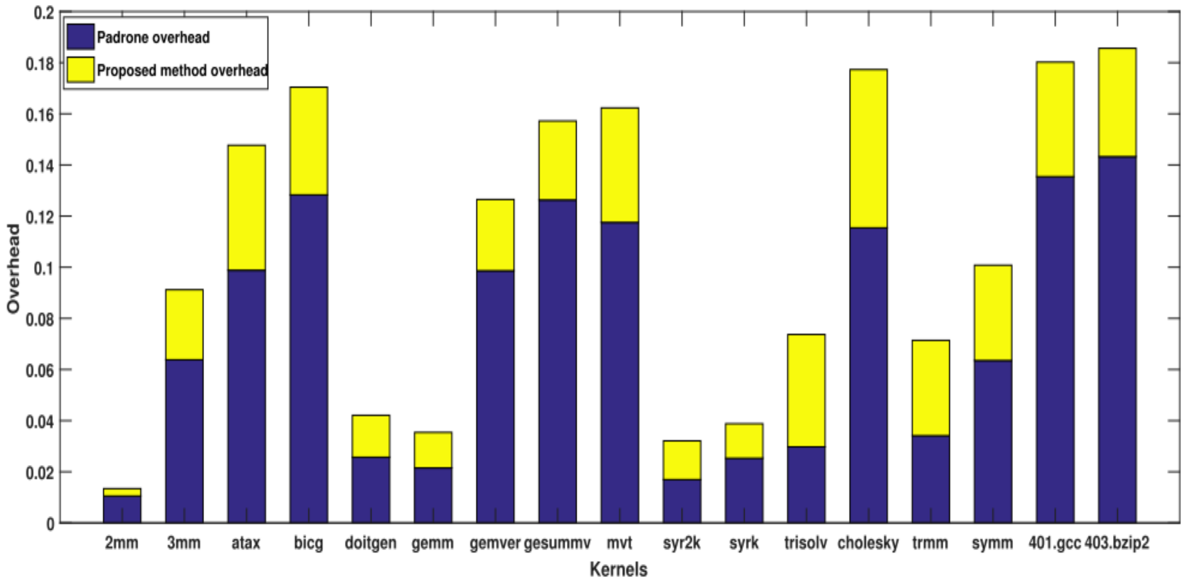

6- The paper mentions speculative execution but does not present any results. Adding this data can demonstrate potential speed up.

- **<u>Response:</u>** Thank you for your valuable note. Our paper main contributions are for dynamic data dependence based on static AI technique. The method is applied for binary code at runtime. Moreover, integrating the system with Padrone framework to get initial performance study. The correctness for the dependence has been addressed according to the original/actual dependence occurred and true/false postives. Furthermore, the overhead, caused by the analysis, has been computed. Therefore, the impact of our applied method has been studied in terms of correctness and overhead. The computed overhead is low and it would be diminished then using the speculative parallelization technique. The impact of speculative parallelization on our analysis would be studied in the future work.
- This concept is clearly explained in the paper at <u>(page 14-15, Section 4.7, lines 608-627)</u> and for easy of your reference, here we mention regarding this comment as follows:

"Parallelization techniques are widely used in almost all devices (computers, smart phones, embedded systems). Speculative parallelization is one of the most challenging techniques that could exploit our proposed method to detect dependent instruction pairs. The technique of Yusuf et. al [34] (subsystem of our full design presented in Fig. 1) proposes a new speculative parallelization technique which relies on our AI dependence approach. This technique utilizes the on-stack replacement to allow forking new process to enter the speculative state and kill the faulty process. The system creates a serial process which would be suspended at each checkpoint to preserve the previous work yet. Simultaneously, the parallel execution of the same program continues till reaching to the next checkpoint which is able to commit the gained work. If there is a violation occurred during runtime the speculative technique has the ability to abort the parallel execution and continue with the serial program which contains all saved work on previous checkpoints. Our system mainly provides the analysis of the dependence at the loop to be

utilized by the speculative system at the current run. It does not require a new run to benefit from our method's result. Therefore, our system contains a dynamic automatic binary-level strong analysis.

In Section 5, we have studied the main metrics affected by our approach of dynamic data dependence. The experiments examines the overhead from the applied dependence analysis. Furthermore, the correctness of the system is examined using extracting the actual dependent pairs and the produced dependent pairs by our technique."

- Also, future work section is updated in (page 18, Section 6, lines 759-769) as follows:

"Future work includes extending the work to consider pointer arithmetic. The work involves also studying more complex kernels with much complicated operations as some of floating point operations and vector instructions. Moreover, our system would be provided with abstracting the dependence distance between iterations. Also, we need to study the function calls handling as well as the dependence analysis during the execution of the function code. The indirections in case of [-inf, inf] would be handled as a future work to correctly get the results. Furthermore, we plan to integrate the dependence analysis work into the sub-system of speculative parallelizing environment. Therefore, we would study the full system behavior and investigate the approach validation. Speculative parallelization would highly reduce the per-computed overhead with more speedup."

Reviewer #2:

This paper describes a new method for extracting data dependence from binary code using abstract interpretation at runtime.

This paper is in general well written and easy to understand. This helps me quickly focus on evaluating the technical work of this paper. I do have three questions that I hope the authors to address:

1- **The evaluations fall short to support the claim by the author that "Our initial results the accuracy of our proposed approach in detecting data dependence dynamically". Even though the table 2 in the paper shows that the proposed approach finds all the actual data dependency (true positives), the paper does not contain any description about false positive. At line 596, the sentence "Moreover, the second column explains the true positives." indicates that table 2 only contains true positive numbers.**

- **Response:** We would like to thank the anonymous reviewer for careful review of our work. The table is updated with true positives and false positives. The false positives are the dependent instructions' pairs which may not be really dependent. The speculator would decide according to the current state of execution whether these instructions would run parallelized or not.
- This is clearly explained in the paper at (page 16, Section 5, lines 666-676) as follows:

“All dependent pairs in Table 2 are between two instructions containing WAR dependence. The true positives, second column, are illustrated as the number of actual dependent pairs. Most of the experimented kernels contains only one dependent pair of instructions with WAR dependence which is included as a *C* equations in the for-loop. However, the kernels *trisolv*, *bicg* and *syr2k* have two *C* statements with dependence in each one. Also, the kernels *cholesky* and *symm* include three dependent between each pair of statements. Hence, our approach will provide the speculative system that each kernel contains dependent pair which cannot be parallelized. Moreover, the false positives, third column, numbers will not affect the speculative parallelization performance. The parallelization decision would be relied on the current state also as well as our dependence analysis.”

- Also, the table is updated with true and false positives in the second and third columns at (page 16, Section 5) as follows:

Table 2. Dependence Detected in Kernels of Polybench by Our Method and Static AI (True and False Positives)

Kernel	True Positives (Our Approach)	False Positives (Our Approach)	True Positives (Static AI)	False Positives (Static AI)
2mm	1	0	1	2
3mm	1	0	1	2
bicg	2	0	2	2
gesummv	1	0	1	1
gemm	1	0	1	2
mvt	1	0	1	2
syrk	1	0	1	1
syr2k	2	0	2	1
atax	1	2	1	2
gemver	1	0	1	1
doitgen	1	2	1	2
trisolv	2	1	2	2
cholesky	3	1	3	2
trmm	1	1	1	2
symm	3	1	3	2

2- The experiments in this paper do not contain comparisons against any baseline. I do understand that it is likely that there is not any previous work on detecting data dependence on binary code. However, there still should be baseline to show the necessity of each component in the proposed approach. For example, I can propose a simple baseline that uses static binary level backward slicing to determine data dependence; such baseline does not incur “runtime” overhead and can be implemented with existing dataflow analysis toolkit, such as the dataflowAPI in Dyninst (github.com/dyninst/dyninst). I do not claim this baseline is better than the proposed approach. However, line 603 to 605 of the paper states that the data dependences are in “a C equation” and “two C statement”. A C equation typically correspond to several adjacent machine instructions; static analysis can extract data dependence for such cases. In other words, while the proposed approach has good potential, the evaluation does not highlight the actual effectiveness of the proposed approach.

- **<u>Response:</u>** Thanks for your valuable suggestion. We have made some investigation about the technique you suggested to compare our proposed technique with. However, due to limited time of this review cycle, we found it is more efficient to compare our method with the traditional static AI dependence technique. We have implemented a baseline technique using the well-known static AI in Padrone framework. This technique is based on acquiring the abstract intervals from the binary code of the extracted hot function. The abstract intervals are computed from the available values stored in registers, types of operations and operators. The arguments of the hot function would not read the current values of them. This would affect the accuracy for the static analysis technique. The traditional static AI is applied in Polybench kernels. The results show that our technique higher correctness. Therefore, the correctness would be higher in all applied kernels. Furthermore, static AI technique may pessimistically handle the case of rare branches. Our technique analyzes the hot trace executing in at runtime.
- This comparison is clearly explained in the paper at <u>(page 16-17, Section 5, lines 677-737)</u> and for easy of your reference, here we mention regarding this comment as follows:

"**Our proposed technique have been examined in comparison with a traditional static analysis technique. This static method is based on computing the static AI intervals at compile-time as well as monitoring the definitions of registers would be used by the binary code. We have implemented a standard static AI technique using Padrone binary modification system. Padrone would profile and extract the hot function, therefore the extracted code would be applied statically to compute data dependence. In each program point, the system checks mainly three parts. Firstly, the type of operation whether it includes write, read,…, etc according to the binary instruction type *mov*, *add*, *sub*,.. etc. Secondly, the types of operators should be determined. Thirdly, the available values, if any, is immediate or not. According to these three parts, the system could compute the abstract interval. Moreover, the system would use previous definitions at each program point. If there are no available values, the abstract intervals would be assumed as [-*inf*, *inf*].**

Table 2. Dependence Detected in Kernels of Polybench by Our Method and Static AI (True and False Positives)

Kernel	True Positives (Our Approach)	False Positives (Our Approach)	True Positives (Static AI)	False Positives (Static AI)
2mm	1	0	1	2
3mm	1	0	1	2
bicg	2	0	2	2
gesummv	1	0	1	1
gemm	1	0	1	2
mvt	1	0	1	2
syrk	1	0	1	1
syr2k	2	0	2	1
atax	1	2	1	2
gemver	1	0	1	1
doitgen	1	2	1	2
trisolv	2	1	2	2
cholesky	3	1	3	2
trmm	1	1	1	2
symm	3	1	3	2

The extracted hot function may include arguments. The static AI analysis is not able to detect the recent values for these arguments which affect the correctness. However, our technique would obtain the values of the arguments during runtime. These values are converted from concrete domain to abstract domain.

The impact of static AI technique is presented in terms of correctness which are shown in Table 2 the fourth and fifth columns include the true and false positives respectively. The false dependent pairs exist in all kernels with at least 1 or 2 pairs. In our method the existence of false positives are limited to number of kernels most of cases are 1 pair except two kernels, *doitgen* and *atax*. Therefore, Table 2 proofs that our method correctness is higher than the applied static AI method.

For more clarification, the following example is supported to investigate the main difference in correctness between our method and static method.

```
int a1[1000] = {0}, a2[1000] = {0}, sum[1000] = {0};
int i = 0;
int c1, c2;
cin >> c1 >> c2;
...
while(i < 1000) {
  if(c1 > c2){                  //S1
  a1[i] += a2[i];               //S2
  sum[i] += a1[i] + a2[i];      //S3
  }
  else if(c1 < c2){             //S4
  sum[i] = a1[i] + a2[i];       //S5
  }
  else{                         //S6
  a2[i] += a1[i];               //S7
  sum[i] += a1[i] + a2[i];      //S8
  }
  i++;
}
```

The loop contains mainly three branches. First branch, *S1*, contains data dependence at *S2* and *S3*. Second branch, *S2* does not contain any data dependence. Last branch, S6 includes data dependence for *S7* and *S8*. The branches rely mainly on the input of variables *c1* and *c2*. One of these branches will be executed during all loop iterations. Static analysis would be applied on the loop for all branches which may be executed or not. If *S4* is true, the other two branches would not be executed till the end of loop. Therefore, the loop would continue execution in parallel. However, static analysis would preserve the other two branches dependence. The speculator would resume the execution serially because of dependent program points which will never be executed. In our method, the analysis would detect that there is one branch is executed at this run. Our dynamic analysis works for the hot trace in the hot function. Therefore, if *S4* branch is taken, the analysis would detect that there is no dependence in the current run. Subsequently, if one of the other branches is taken, our analysis would detect only the dependence in the taken branch. The other branches would not be analyzed which increase our technique's correctness.”

3- In Section 4.3.2 Abstract Operations, the authors do not discuss how to handle function calls. The hot function can contain a loop in which multiple small functions are called. It is not clear to me how the proposed approach can be applied to this case.

- **Response:** Thank you for giving us a chance to clarify this issue. The function calls are not part of our main target. The function call requires some complicated requirements which are still not-solved problem in parallelization. Therefore, our system terminates the analysis once there is a function call detected. Thus, we have added this point as a future work.
- Also, the manuscript is updated in (page 9, Section 3, lines 383-391) as follows:

“The hot function which contains the hot loop may include function call(s). These function calls are not studied or handled in our method because each function call needs complex requirements which are not solved in purpose of parallelization [28]. Once our system detects the function call, then the analysis would terminate.”

- Also, the future work is explained in (page 18, Section 6, lines 759-769) as follows:

“Future work includes extending the work to consider pointer arithmetic. The work involves also studying more complex kernels with much complicated operations as some of floating point operations and vector instructions. Moreover, our system would be provided with abstracting the dependence distance between iterations. Also, we need to study the function calls handling as well as the dependence analysis during the execution of the function code. The indirections in case of [-inf, inf] would be handled as a future work to correctly get the results. Furthermore, we plan to integrate the dependence analysis work into the sub-system of speculative parallelizing environment. Therefore, we would study the full system behavior and investigate the approach validation. Speculative parallelization would highly reduce the per-computed overhead with more speedup.”

Finally, the authors cannot thank the reviewers and the associate editor enough for their keen observations and valuable guidance. It’s really appreciated.

Best Regard.
