# Supplementary material for: Binary-level data dependence analysis of hot execution regions using abstract interpretation at runtime
Source: PLoS One. 2020 Apr 9;15(4):e0230904. doi: 10.1371/journal.pone.0230904 (PMC7144976; doi:10.1371/journal.pone.0230904)
Supplement: Attachment — Submitted filename: Responses to Reviewers.pdf [file pone.0230904.s002.pdf]

Reply letter to the reviewer comments on the paper titled

“Binary-level data dependence analysis of hot execution regions using abstract interpretation at runtime”

First of all, we would like to express our gratitude to the associate editor and anonymous reviewers for their careful reviews and valuable suggestions. The comments are well taken and, accordingly, the manuscript has been revised carefully. Reviewers’ comments are represented using **Bold Red Font.** All revisions in the manuscript have been marked with **Bold Blue Font** color. Our point-to-point responses to the comments are listed as follows.

Responses to Reviewer

Reviewer #1:

1- **I do not agree with the statement "Moreover, the false positives, third column, numbers will not affect the speculative parallelization performance. The parallelization decision would be relied ".on the current state also as well as our dependence analysis**

If the parallelization decision relies on the dependent analysis, then false positives of the dependent analysis will lead to missed parallelization decision. Let me use an extreme example to explain. Suppose I build a dependent analysis where one instruction depends on every other instruction. This analysis will not miss any true dependency (no false negative), but cause a ton of false positive. I do not think we can make any useful parallelization decision based on such .analysis with tons of false positive

The authors provide the results showing that their approach will lead to fewer false positives compared to a base line, so false positive is a not problem for the paper. I think the false positive results improve the paper. However, I think the authors should rewrite the sentences about false positives. I disagree that "false positives will not affect the speculative ."parallelization performance

- **Response:** We would like to thank the anonymous reviewer for careful review of our work. Your comment is totally correct. False positives affect the performance because these cases would let the parallelization technique misses the opportunity to parallelize the code. Therefore, we have updated the revised manuscript.
- This is clearly explained in the paper at (page 16, Section 5, lines 661-671) and for easy of your reference, we write it down here:

“Moreover, the second column explains the true positives. This column's presented numbers are the dependent instructions pairs which are correctly dependent. The third column presents the false positives number of produced dependent instructions pairs which may not be dependent. If the false positives' values equal 0, thus the parallelizer would take the right decision to parallelize or not according to the true positives' values only. Apparently, the result of false positives is able to reflect the efficiency of any proposed approach. However, the false positives non-zero values will affect this efficiency

partly. The speculator would take the decision during execution according to the preserved results of the dependence flags map. Our results prove the accuracy of our proposed approach in detecting data dependence dynamically."

- Also, this part is highlighted in the modified manuscript as shown at (page 5, Section 16, lines 679-683) as follows:

"Moreover, false positives, third column numbers present low values in comparison with static false positives. Therefore, the missed opportunities of speculative parallelization will be lower and the accuracy will be higher than the static version. The parallelization decision would be relied on the current state as expected by our dependence analysis."

2- I appreciate the authors for adding a strong baseline into the results and add a detailed examples for comparing the baseline with their approach. This definitely helps understanding the strength of the authors' approach. My honest question is in Table 2, both the authors' approach and the baseline find all the true positives in all the benchmarks used by the authors. I am not surprised by the effectiveness of the baseline approach because the authors have stated that the data dependences are in "a C equation" and "two C statements". So, the "true positives" results do not show that authors' new approach is better than the baseline. The authors' new approach is much better for false positives compared to the baseline, but the .current revision seems to downplay the importance of false positives

Therefore, I think the authors either need to find more benchmarks where the new approach finds more true positives compared to the baseline, or clearly explain the importance of false .positives. Of course, the best scenario is that the authors can do both

- **Response:** Thank you for giving us a chance to clarify this issue. False positives would affect in case of existing of possibly parallelize code. The speculator may have a decision of not to parallelize, however the code could be parallelized and the detected dependence is false. This problem will miss opportunities to run the program in more efficient way. According to the results, our method mostly includes zero false positives. Moreover, the non-zero false positives are less than the static approach's false positives.
- We have addressed this comment in the revised manuscript by adding few sentences in (page 16, Section 5, lines 661-671) and for easy of your reference, we write it down here:

"Moreover, the second column explains the true positives. This column's presented numbers are the dependent instructions pairs which are correctly dependent. The third column presents the false positives number of produced dependent instructions pairs which may not be dependent. If the false positives' values equal 0, thus the parallelizer would take the right decision to parallelize or not according to the true positives' values only. Apparently, the result of false positives is able to reflect the efficiency of any proposed approach. However, the false positives non-zero values will affect this efficiency partly. The speculator would take the decision during execution according to the

preserved results of the dependence flags map. Our results prove the accuracy of our proposed approach in detecting data dependence dynamically."

- Also, this response is discussed in the paper at (page 5, Section 16, lines 679-683) as follows:

"Moreover, false positives, third column numbers present low values in comparison with static false positives. Therefore, the missed opportunities of speculative parallelization will be lower and the accuracy will be higher than the static version. The parallelization decision would be relied on the current state as expected by our dependence analysis."

3- I am fine with putting handling of function calls in future work, which is indeed a difficult problem.

- **Response:** Thank you for your comment. Your revision comments give us a chance to improve the manuscript.

Reviewer #3:

1- The paper is well written and has a good structure and organization. The Authors clearly show .the limitations of current related work and the contributions of their proposal in the paper

- **Response:** We would like to thank the anonymous reviewer for careful review of our work.

2- The authors adequately answered the questions raised by the reviewers in the revised manuscript. However, the authors need to proofread the paper for grammar in the final submission.

- **Response:** Thank you for your valuable comment. We have revised the paper in order to improve the proofreading and grammar.

Finally, the authors cannot thank the reviewers and the associate editor enough for their keen observations and valuable guidance. It's really appreciated.

Best Regard.
